# Signs of Death by Drowning

**Published:** 1880-03-20

**Authors:** 


### *SIGNS OF DEATH BY DROWNING.*

In the *Annales d'Hygiene et de Medecine Legale*, Drs. Bergeron and Montano give the following signs as establishing death by drowning :

1. The presence of frothy foam, not only in the pharynx and the larynx, but also in the bronchi, is the constant sign of death by submersion, whether syncope or asphyxia predominated in the mode of death, and whether the individual was free in his movements or was thrown into the water after having been made insensible by opium or chloroform, or was partly suffocated, or was fettered in his action. This absolute constancy of the presence of foam, whatever the special condition in which the submersion occurred, is, in the opinion of the authors, the single sure uniform sign proving death by drowning.

2. There is always a certain degree of congestion, and sometimes sub-pleural ecchymoses are seen; but these ecchymoses, which give the lungs a spotted or speckled look, are unlike the punctate ecchymoses of suffocation.

3. The intensity of hyperæmia and the extent of the ecchymoses are always in proportion to the efforts of the animal while struggling against submersion. It is the same also with the human subject, as has been verified in all necropsies made by the authors at the morgue in Paris during the last ten years. This fact permits one at a necropsy to learn concerning what passed in the last moments of life, to know whether or not the individual struggled long and vigorously during the act of drowning.—*Med. Reporter.*

